# Maternal and perinatal characteristics and outcomes of pregnancies complicated with COVID-19 in Kuwait

**DOI:** 10.1186/s12884-020-03461-2

**Published:** 2020-12-02

**Authors:** Amal Ayed, Alia Embaireeg, Asmaa Benawadh, Wadha Al-Fouzan, Majdeda Hammoud, Monif Al-Hathal, Abeer Alzaydai, Ashraf Ahmad, Mariam Ayed

**Affiliations:** 1 Obstetrics and Gynecology Department, Farwaniya Hospital, Sabah Al Nasser, Kuwait; 2 Paediatric Department, Farwaniya Hospital, Sabah Al Nasser, Kuwait; 3 Department of Microbiology, Faculty of Medicine, Kuwait University, Jabriya, Kuwait; 4 Paediatrics Department, Faculty of Medicine, Kuwait University, Jabriya, Kuwait; 5 Neonatal Department, Maternity Hospital, Kuwait City, Kuwait; 6 Obstetrics and Gynaecology Department, Al-Adan Hospital, Hadiya, Kuwait; 7 Neonatal Department, Farwaniya Hospital, Subah An Nasser, 81400 Kuwait City, Kuwait

**Keywords:** SARS-CoV-2, COVID-19, Pregnant women, Neonates, Clinical features

## Abstract

**Background:**

The effect of SARS-CoV-2 infection in pregnant women and newborns is incompletely understood. Preliminary data shows a rather fluctuating course of the disease from asymptomatic or mild symptoms to maternal death. However, it is not clear whether the disease increases the risk of pregnancy-related complications. The aim of the study is to describe the maternal and neonatal clinical characteristics and outcome of pregnancies with SARS-CoV-2 infection.

**Methods:**

In this retrospective national-based study, we analyzed the medical records of all pregnant women infected with SARS-CoV-2 and their neonates who were admitted to New-Jahra Hospital (NJH), Kuwait, between March 15th 2020 and May 31st 2020. During the study period and as part of the public health measures, a total of 185 pregnant women infected with SARS-CoV-2, regardless of symptoms, were hospitalized at NJH, and were included. Maternal and neonatal clinical manifestations, laboratory tests and treatments were collected. The outcomes of pregnancies included miscarriage, intrauterine fetal death (IUFD), preterm birth and live birth were assessed until the end date of the outcomes follow-up (November 10th 2020).

**Results:**

A total of 185 pregnant women infected with SARS-CoV-2 were enrolled with a median age of 31 years (interquartile range, IQR: 27.5–34), and median gestational age at diagnosis of SARS-CoV2 infection was 29 weeks (IQR: 18–34). The majority (88%) of these women had mild symptoms, with fever (58%) being the most common presenting symptom followed by cough (50.6%). At the time of the analysis, out of the 185, 3 (1.6%) of the pregnant women had a miscarriage, 1 (0.54%) had IUFD which was not related to COVID-19, 16 (8.6%) had ongoing pregnancies and 165 (89%) had a live birth. Only 2 (1.1%) of these women developed severe pneumonia and required intensive care. A total of 167 neonates with two sets of twins were born with median gestational age at birth was 38 (IQR: 36–39) weeks. Most of the neonates were asymptomatic, and only 2 of them tested positive on day 5 by nasopharyngeal swab testing.

**Conclusions:**

In this national-based study, most of the pregnant women infected with SARS-CoV-2 showed mild symptoms. Although mother-to-child vertical transmission of SARS-CoV-2 is possible, COVID-19 infection during pregnancy may not lead to unfavorable maternal and neonatal outcomes.

**Supplementary Information:**

**Supplementary information** accompanies this paper at 10.1186/s12884-020-03461-2.

## Background

In late December of 2019, a novel coronavirus (SARS-CoV-2) emerged in the Chinese city Wuhan was identified among a cluster of pneumonia cases [1]. The outbreak of coronavirus disease (COVID-19) rapidly spread to become a global pandemic as declared by the World Health Organization in March 2020 [1–3]. This remarkable widespread SARS-CoV-2 reflects a new public health crisis [4].

SARS-CoV-2 is a member of the Coronavirus family, and other pathogens from this family have inflicted a range of viral infections, including Middle East Respiratory Syndrome (MERS) and Severe Acute Respiratory Syndrome (SARS) [5]. The main route of transmission is through respiratory droplets and direct contact. While the spectrum of the disease severity ranges from mild to critical, most are mild. Most of the fatal cases occurred in patients with advanced age or underlying comorbidities [6]. However, to this date, the SARS-CoV-2 mortality rate is greater than MERS and SARS combined and has a global Case Fatality Rate of about 6.4% [1].

Although it is known that pregnant women have a reduced immunity [5], and they are at risk for COVID-19 infection during the current pandemic, it is not clear how the disease manifestation would be different in pregnant women from non-pregnant women. It is known that the body’s immune system and response to viral infections might be changed due to pregnancy, which explains the cause of more severe symptoms [7]. However, some previous studies have shown that pregnant women are not more susceptible to SARS-CoV-2 than non-pregnant women [8–11]. Considering the severity of disease, limited data suggest that the pregnant women may present with severe symptoms that include hypoxia, hypotension, electrolyte disturbances, and placental hypo perfusion, which can provoke fetal distress, preterm labor, miscarriage, or fetal death [12–18].

Preliminary evidence from the diseases caused by the H1N1 virus (H1N1 influenza) and other coronaviruses (SARS and MERS) have suggested that pregnant women are more likely to have severe manifestations of the disease, morbidity, and mortality as compared to non-pregnant women [19–22]. Moreover, some studies have shown increased risk of these critical illness with the increased duration of pregnancy [23–25].

To date, information about the different routes (transplacental, perinatal, intrapartum, and postnatal) and risks of transmission of COVID-19 to newborn infants is based on case series [13]. In consideration of the fact that SARS-CoV-2 is a respiratory virus, the risk of vertical transmission is supposed to be very low or absent. This supposition is supported by the evidence that no vertical transmission was detected in other members of the coronavirus family (SARS and MERS) [19].

The risk of perinatal transmission of SARS-CoV-2 is unknown. Additionally, since the risk of postnatal transmission remains to be elucidated, it is debatable whether the newborn should be immediately separated from the COVID-19 positive mother [8]. A study comprised of outcomes of 7 newborns concluded that COVID-19 positive mother does not cause unfavorable outcomes in their neonates, but it is required to separate newborns from mothers right away to avoid potential threats of SARS-CoV-2 infection [26]. In another study, contrary to the previous one, it is concluded that separation may not prevent infection, and early separation might increase neonatal risk of pneumonia [27]. However, the severity of postnatally acquired disease in the newborn is unknown. A case series of 10 neonates born to COVID-19 positive mothers reported no evidence of vertical transmission of SARS-CoV-2. However, it suggested that perinatal SARS-CoV-2 infection may cause fetal distress, premature labor, respiratory distress, thrombocytopenia accompanied by abnormal liver function, and even death among neonates [12].

We believe that the information on outcomes of such pregnancies could continuously update guidance and management procedures for COVID-19 positive pregnant mothers and their neonates. Therefore, the study aimed at describing the maternal as well as neonatal clinical features and outcome of pregnancies complicated with SARS-CoV-2 infection.

## Methods

### Study design

We performed a retrospective national-based analysis of medical records from all SARS-CoV-19 positive pregnant women and their neonates who were admitted to New-Jahra Hospital, Kuwait between March 15th, 2020 and May 31st, 2020, and the outcomes were assessed till the end date of follow-up (November 10th, 2020). New Jahra hospital was designated as a national hospital for the management of COVID-19 infected pregnant women and neonates born to infected mothers in Kuwait. During the study period, as part of Kuwait’s national COVID- 19 policy, all pregnant women were tested if they had SARS-CoV-2 symptoms or had been in contact with infected patients. Moreover, all confirmed COVID-19 positive pregnant women were admitted to the maternity department regardless of symptoms or gestational age at diagnosis of SARS-CoV-2 infection for at least 14 days or till resolution of symptoms. Patient management was based on local and international protocols (Additional file 1).

The study was approved by the Ethics Committee of the Ministry of Health of Kuwait (2020/1420).

### Patient selection criteria

We enrolled all pregnant women admitted to the maternity department due to SARS-CoV-2 infection, as confirmed by real-time reverse transcriptase-polymerase chain reaction (RT-PCR) assay of nasopharyngeal swab specimens (Cobas 6800 Systems, Roche, Switzerland)/(TaqPath, Thermo-Fisher Scientific, USA). Patients who had equivocal or negative testing results were excluded from the study.

### Assessment of neonates

A little was known about the risk of postnatal acquired COVID-19 disease during the early pandemic. However, our national policy recommended that all neonates born to SARS-CoV-2 infected mother should be assessed by the neonatal team after birth. They were isolated immediately and admitted to a designated neonatal intensive care unit. All neonates born to SARS-CoV-2 positive mothers were admitted to a negative pressure isolated single room, cared for by health care providers wearing full personal protective equipment, and were fed either breast milk or artificial formula. Moreover, nasopharyngeal swab testing was performed for all neonates on day 5 and then on day 14. Since June 2020, the national policy has been updated and does not recommend immediate separation of the newborn and encourage immediate skin to skin contact if mother and baby are well.

### Data collection

Maternal and neonatal data were extracted from the electronic medical records or medical charts using a standardized data collection sheet. For maternal data, we recorded the information on gestational age at diagnosis of SARS-CoV-2, parity (nulliparous or multiparous), pre-existing medical condition (i.e., including hypertension, diabetes, bronchial asthma, hypothyroidism, and autoimmune diseases) and presenting symptoms prior to admissions, such as fever, sore throat/ nasal or sinus congestion, cough/chest pain, vomiting/diarrhea, and fatigue/myalgia). Information on current pregnancy was also collected, including gestational diabetes, pregnancy-induced hypertension, pre-eclampsia, and multiple pregnancies. We collected the laboratory and chest x-ray results upon admission to the hospital. Information on patient-specific therapies, such as antenatal steroids for lung maturation, administration of antibiotics, antivirals, and thromboprophylaxis (i.e. low molecular weight heparin) was also collected. For patients admitted to intensive care unit, we obtained data on maximum respiratory support (i.e. intubation and mechanical ventilation), acute respiratory distress syndrome (ARDS) as defined according to the Berlin criteria and duration of ventilation [28].

For neonatal data, we collected gestational age at delivery, mode of delivery, birth weight, 1- and 5-min Apgar score, and the results of nasopharyngeal swabs. Data entry and quality were checked and reviewed independently by two investigators (AE and AB).

We defined gestational age as the number of weeks calculated according to the last normal menstrual period or expected date of delivery according to an early ultrasound scan. Gestational diabetes was defined as high blood sugar (glucose) using the Oral Glucose Tolerance Test as a screening method (fasting blood sugar ≥5.1 mmol/L or 1 h postprandial ≥10 mmol/L or 2 h postprandial ≥8.5 mmol/L) that developed during pregnancy and usually disappeared after giving birth. Preeclampsia was defined as new onset of hypertension (over 140 mmHg systolic or over 90 mmHg diastolic) after 20 weeks of pregnancy and significant proteinuria. Pregnancy-induced hypertension (PIH) was defined as a new hypertension presenting after 20 weeks of pregnancy without significant proteinuria.

### Statistical analysis

We reported continuous variables as mean ± standard deviation (SD) or median (interquartile range [IQR]) and categorical variables as number and percentage. Statistical analysis was performed using STATA/IC 14 software (STATA Corp, College Station, Texas).

## Results

During the study period, 185 pregnant women were identified with confirmed SARS-CoV-2 infection with a median age of patients was 31 years (IQR: 27.5–34), and 19.5% were nulliparous. The majority of patients (91%) had no pre-existing medical comorbidities, 19 (10.3%) patients had gestational diabetes, and 4 (2.3%) had PIH. The median gestational age at diagnosis of SARS-CoV-2 was 29 weeks (IQR: 18 to 34), and half of the women were in their 3rd trimester. Additionally, the majority of the patients (88.6%) were symptomatic. The most common symptom was fever in 105 (58%) patients, followed by cough in 90 (50.6%) patients, and the median duration of symptoms prior to hospital admission was 2 days (IQR:1–4). Table 1 presents the characteristics of pregnant women with confirmed SARS-CoV-2 infection.

**Table 1:** Characteristics of pregnant women with confirmed SARS-CoV-2 infection

Characteristic	Total number = 185 (%)
Age, years (median, IQR)	31 (27.5–34)
Parity:	
Nulliparous	36 (19.5%)
Multiparous	149 (80.5%)
Pre-existing medical disease:	
Hypothyroidism	7 (3.9%)
Bronchial Asthma	4 (2.2%)
Chronic diabetes	1 (0.5%)
Others (rheumatoid arthritis n = 1, anxiety n = 1, peptic ulcer = 1, *n* = 1, Crohn’s disease, n = 1, beta-thalassemia minor = 1)	5 (2.8%)
Missing data	5 (2.8%)
Gestational diabetes	19 (10.3%)
Preeclampsia	4 (2.3%)
Gestational Age at diagnosis of SARS-CoV-2 infection weeks (median, IQR)	29 (18–34)
Trimester:	
1st (less than 12 weeks)	21 (11.4%)
2nd (13–27 weeks)	64 (34.6%)
3rd (> 28 weeks)	95 (51.3%)
Postpartum	5 (2.7%)
Multiple pregnancies	2 (1.1%)
Symptoms:	165
Fever	105 (58%)
Cough	90 (50.6%)
Loss of smell	10 (5.8%)
Sore throat /rhinorrhea	42 (24.3%)
Malaise/Fatigue	26 (15.1%)
Chest pain/shortness of breath	22 (12.8%)
Vomiting/diarrhea	11 (6.4%)
Duration of symptoms prior to admission, days	2 (1–4)
Asymptomatic	21 (11.3%)

The laboratory findings of pregnant women are shown in Table 2. Seventeen (9.2%) of the pregnant patients had leukopenia, and 29 (15.7%) had lymphopenia. Forty-six (37%) patients had elevated C-reactive protein and 12 (11%) had elevated procalcitonin. Elevated alanine aminotransferase (ALT), aspartate aminotransferase (AST), D-dimer and lactate dehydrogenase (LD) were observed in 24.2%, 11.2, 10 and 42%, respectively of the patients in which the test was ordered. Eight (15.4%) patients had a chest x-ray diagnosis of pneumonia.

**Table 2:** Laboratory and radiological characteristics of pregnant women

Laboratory test	Laboratory value	
	N	Median (IQR) N (%)
White blood count (WBC) X10^9^per L	185	6.7 (5.6–9.2)
WBC Less than 4		17 (9.2%)
Lymphocytes X10^9^/L	185	1.5 (1.1–2.05)
Lymphocyte less than 1		29 (15.7%)
Lymphocyte more than 3.6		32 (17.3%)
Neutrophils X10^9^	185	4.8 (3.5–6.8)
Neutrophils less than 1.8		14 (7.6%)
more than 7.1		64 (34.6%)
Platelets X10^9^	185	214 (178–252)
Platelets Less than 100		2 (1.1%)
C-reactive protein (CRP) (mg/L)	124	4.5 (1.1–14.6)
CRP > 8		46 (37.1%)
Procalcitonin (PCT) (ng/mL)	108	0.05 (0.05–0.08)
PCT > 0.2		12 (11.1%)
Aspartate aminotransferase (AST) (U/L)	125	21 (16–28)
AST more than 40		14 (11.2%)
Alanine aminotransferase (ALT) (U/L)	149	13 (11–24)
ALT more than 40		36 (24.2%)
Lactate dehydrogenase (LDH) (IU/L)	121	181 (154–222)
LDH more> 200		51 (42.1%)
D-Dimer (ng/mL)	100	488.5 (333–754.5)
500–1000		6 (6%)
> 1500		4 (4%)
Albumin (g/L)	148	32 (30–35)
Albumin less than 30		31 (20.9%)
Chest x-ray diagnosis of pneumonia	52	8 (15.4%)

Maternal treatment and outcomes are shown in Table 3. Two (1.1%) patients required critical care for worsening respiratory conditions. The majority (97.3%) of the patients received Oseltamivir, and only 2 (1.1%) received Lopinavir-ritonavir. Ceftriaxone was given in 24.3% of the patients, and 96.7% received low molecular weight heparin.

**Table 3:** Maternal hospital treatment and outcomes

Characteristics	Number (%) of patients
Drug therapy:	
Oseltamivir	180 (97.3%)
Lopinavir-ritonavir	2 (1.1%)
Ceftriaxone	45 (24.3%)
Azithromycin	5 (2.7%)
low molecular weight heparin	179 (96.7%)
Antenatal steroid for lung maturation	38 (20.5%)
Intensive care unit (ICU) admission	2 (1.1%)
ECMO	0
Final outcomes:	
Died	0
Discharged	185 (100%)
Length of hospital stay, days (median, IQR)	15 (14–22)

ECMO; extracorporeal membrane oxygenation

Two patients requiring Intensive care unit (ICU) were at 37- and 38-weeks’ gestation. One developed hypoxia (oxygen saturation, 88%) and shortness of breath due to worsening pneumonia necessitating intubation and emergency caesarian-section, while the second patient had worsening respiratory condition 24 h post-delivery (Table S1). None of them required extracorporeal membrane oxygenation (ECMO), and both got discharged subsequently. At the time of follow-up, all of the patients were discharged, and the median duration of hospital stay was 15 days (IQR:14–22).

Pregnancy and neonatal outcomes are shown in Table 4. At the time of the analysis, out of the 185, 16 (8.6%) of the women had ongoing pregnancies, 165 (89%) had a live birth, 3 (1.6%) had a miscarriage, and 1 (0.54%) had intrauterine fetal death, which was not related to COVID-19. Details of the cases resulting in spontaneous miscarriages are shown in Tables S2.

**Table 4:** Pregnancy and neonatal outcomes

Outcomes	Number (%) of patients
Pregnancy outcomes:	
Continued pregnancy	16 (8.6%)
Miscarriage	3 (1.6%)
Intrauterine fetal death (IUFD)	1 (0.54%)
Delivered live birth (including two twin birth)	165 (89%)
Gestational age at delivery, weeks	
Median (interquartile range)	38 (36–39)
27–32^+ 6^ weeks	2 (1.2%)
33–36^+ 6^ weeks	42 (25.5%)
≥ 37 weeks	121 (73.3%)
Mode of delivery	
C-section	79 (47.8%)
Elective (repeat/maternal request)	35 (44.3%)
Emergency	10 (12.6%)
Maternal Hypoxia	1 (1.2%)
Preeclampsia	11 (13.9%)
Failed induction/ failure to progress	13 (16.4%)
Fetal distress	9 (11.4%)
Type of anesthesia during C-section	
• General	9 (11.4%)
• Spinal	70 (88.6%)
Vaginal delivery	86 (52.1%)
Instrumental delivery	0
Prolonged rupture of membrane (PROM> 18 h)	6 (3.6%)
Birth weight (grams), median (IQR)	3006 (2750–3215)
Head circumference (cm), median (IQR)	33 (32–34.5)
1-min Apgar score, mean ± (SD)	8 (1)
5-min Apgar score, mean ± (SD)	9 (1)
Neonatal death	0
Neonates tested for SARS-CoV-2 infection	
Positive for COVID-19	
None	165 (98.8%)
Day 5 swab positive test	2 (1.2%)
Day 14 swab positive	1 (0.6%)

The median gestational age at birth was 38 (IQR: 36–39) weeks. Of the 165 women who had given birth, 44 (26.6%) gave birth preterm. One preterm birth at 27 weeks was iatrogenic (due to pathological cardiotocography) and one at 31 weeks due to premature rupture of membrane. Forty-two women gave birth between 33 and 36 weeks, either spontaneous delivery or due to other obstetric causes.

A total of 167 neonates were delivered with two sets of twins. Seventy-nine (47.8%) of neonates were born by cesarean section. Of these, one of the neonates was delivered by emergency cesarean due to maternal hypoxia as a result of worsening pneumonia, and the other 78 were due to either fetal distress or obstetrical indication. Median birth weight was 3006 g (IQR: 2750–3215), and only 5 (3%) neonates required intubation and surfactant due to hyaline membrane disease of prematurity. Two (1.2%) of the neonates tested positive for SARS-CoV-2 infection, and there was no neonatal death.

Clinical characteristics of SARS-CoV-2 positive neonates are shown in Table S3. The first positive neonate was born at 31 weeks by vaginal delivery; the mother presented with preterm premature rupture of membrane and developed intrapartum fever (38.2 °C). The obstetrical cause of fever was ruled out, and the COVID-19 was suspected that was later confirmed on RT-PCR. The second positive neonate was born at 39 weeks’ gestation by spontaneous vaginal delivery after 24 h of premature rupture of membrane. One newborn was asymptomatic from COVID-19 symptoms. The second newborn developed tachypnoea and increase work of breathing on day 4 of age and required humidified high flow nasal cannula for 5 days. The neonate’s clinical condition improved, and a chest x-ray showed complete resolution of the bilateral streaky lung infiltrate.

## Discussion

This is a descriptive study on the maternal as well as neonatal clinical features and outcome of pregnancies complicated with SARS-CoV-2 infection. Thus far, the majority of the preliminary research articles report a small sample size [12, 13, 15, 18]. This study is among the first to report COVID-positive maternal and neonatal clinical characteristics and outcomes with a comparatively large sample size.

In this study, 185 pregnant women were identified with confirmed SARS-CoV-2 infection with. Around 88% were symptomatic, with the most common symptom being fever in 58% of patients followed by cough in 50.6% of patients. These findings are in accordance with a number of preliminary studies [14, 15, 18, 29]. A prospective cohort study using the United Kingdom Obstetric Surveillance System (UKOSS) found fever and cough as common symptoms in pregnant women having the diagnosis COVID-19 disease [29]. A systematic review revealed that the most dominant initial symptoms in pregnant women with COVID-19 were fever and cough [18]. Similarly, a retrospective study found that common symptoms in pregnant women in the wake of SARS-CoV-2 infection are cough and fever, and less common symptoms include shortness of breath, diarrhea, and myalgia [13]. On the contrary, a study found the majority of women being asymptomatic and afebrile at presentation [17]. However, the majority of the studies have supported the evidence of fever and cough as the most common symptoms [14, 15, 30–35].

Data from the preliminary studies found that majority of pregnant women with COVID-19 were in the late second or third trimester [29]. In our study, the median gestational age on diagnosis was 29 weeks, and half of the women were in their 3rd trimester. Likewise, a prospective cohort study using UKOSS found that most women were hospitalized in the 3rd trimester [8, 29]. Moreover, in our study, 10% of the patients had gestational diabetes. Evidence of an association between gestational diabetes and pregnant women infected with SARS-CoV-2 is well documented [29].

With reference to the laboratory findings, in our study, 15.7% of the pregnant patients had lymphopenia, and elevated ALT and AST were observed in 24.2 and 11.2% of the patients, respectively, and 10% had elevated D-dimer. Several studies have reported lymphopenia in both the general population affected by COVID-19 [36] and also in pregnant women affected by COVID-19 [17, 37]. Similarly, a retrospective study found the likelihood of lymphopenia as well as increased concentrations of ALT or AST as one of the clinical manifestations [13]. Pregnancy is widely accepted as a hypercoagulable state [38], and monitoring of D-dimer is also necessary. A case series study has reported elevated D-dimer in pregnant women with COVID-19 and its association with increased mortality rate [39].

Preliminary studies on pregnancies complicated with SARS-CoV-2 infection suggested a low rate of admission to the ICU, maternal, and neonatal death [40]. Moreover, the rate of severe pneumonia in pregnant women was also not greater than the general population [41]. In our study, 15.4% had a radiological diagnosis of pneumonia, and only 2 patients required critical care for worsening respiratory conditions, which are in accordance with the preliminary records. A case series published by the clinicians in New York reported a similarly low rate of around 5% of the pregnant patients with SARS-CoV-2 infection requiring critical care [14]. Likewise, a prospective cohort study using UKOSS reported that one woman per 2400 giving birth required critical care, and the maternal mortality rate was roughly about one in 18,000 women undergoing delivery [29].

The rates of ICU admission and mortality among pregnant women admitted to hospital with COVID-19 are proportional to the rates among the general population [29, 41]. In our study, we found favorable outcomes among pregnant women with SARS-CoV-2 infection. Most of the patients had mild symptoms, with only a few ICU admissions and zero mortality. The clinical features of pregnant women with SARS-CoV-2 infection were similar to those of the general population having SARS-CoV-2 infection, as reported in previous studies [42].

In our study, 26.6% of neonates were born preterm, and 47.8% of neonates were delivered by cesarean section. These findings are supported by similar findings in the past, in the data from the UKOSS study, where 59% of the neonates had cesarean births, and 27% had preterm births [29]. Another study reported that the majority of pregnant women had a cesarean section planned delivery to prevent neonatal transmission of the virus [34]. Similarly, a systematic review finds out that the frequent mode of delivery in cases reported was cesarean section [18].

Newborns born to SARS-CoV-2 infected mothers are most likely to be asymptomatic [12]. Similarly, in our study, 73.3% of the neonates, born full-term, were asymptomatic. The findings of our study propose that neonatal outcome is reassuring, keeping with other studies [29, 43–46]. However, in our study, two neonates tested positive for COVID-19 on day 5 of nasopharyngeal swab testing. According to Shah’s classification, the diagnosis of congenital or intrapartum infection could not be confirmed [47]. Some studies from the past reported inconsistency with no evidence of vertical transmission [13, 34]. Nevertheless, vertically acquired infection is still reasonable and cannot be ruled out as supported by preliminary studies and emerging evidence [8]. Further investigations around the vertical transmission of SARS-CoV-2 infections are needed.

In our study, the mothers of two COVID-19-positive neonates presented with preterm premature rupture of membrane (PROM). PROM is known to be linked to neonatal sepsis, and duration of PROM and viral infection in neonate, especially with herpes simplex and HIV, is well documented, however, it is difficult to make a definitive conclusion here [48, 49].

A major strength of this study is its large sample size, whereas preliminary studies [12, 15, 18] were comprised of small sample size of 10–84 pregnant women and could not draw a firm conclusion. The findings of the study may aid clinicians and patients in the effective management of the pregnancies complicated with SARS-CoV-2 infection. However, there are also some inherent limitations to this study due to its retrospective study design.

## Conclusions

In this national-based study from Kuwait, the majority of the pregnant women with SARS-CoV-2 infection had mild symptoms with no severe illness. Mother-to-child vertical transmission of SARS-CoV-2 is possible; however, the impacts of COVID-19 infection during pregnancy on maternal and neonatal outcomes do not appear to be unfavorable. Further studies are required to evaluate long-term outcomes and potential vertical transmission of SARS-CoV-2 to infants.

## Supplementary Information

**Additional file 1.** Management of patients with COVID-19.

**Additional file 2: Table S1.** Clinical features of pregnant patients with COVID-19 in ICU. **Table S2.** Clinical features of pregnant COVID-19 patients who had a miscarriage. **Table S3.** Clinical features of COVID-19 positive neonate.

## Data Availability

The datasets used and/or analyzed during the current study are available from the corresponding author on reasonable request.

## Abbreviations

RT-PCR: Real-time reversetranscriptasepolymerasechainreaction
MERS: Middle East Respiratory Syndrome
SARS: Severe Acute Respiratory Syndrome
ARDS: Acute respiratory distress syndrome
qSOFA: Quick Sequential Failure Assessment
CRP: C-reactive protein
PIH: Pregnancy-induced hypertension
ICU: Intensive care unit
PROM: Premature rupture of membrane
