# Supplementary material for: Maternal and perinatal characteristics and outcomes of pregnancies complicated with COVID-19 in Kuwait
Source: BMC Pregnancy Childbirth. 2020 Dec 2;20:754. doi: 10.1186/s12884-020-03461-2 (PMC7709095; doi:10.1186/s12884-020-03461-2)
Supplement: Supplementary file 1 — Additional file 1. Management of patients with COVID-19. [file 12884_2020_3461_MOESM1_ESM.pdf]

Management of Hospitalized Adult Patients with COVID-19 Pneumonia

Interim guidance: April 4th 2020 (version 4.1.2)

Clinical syndromes associated with 2019-nCoV infection[1]

- Uncomplicated illness: Patients with uncomplicated upper respiratory tract viral infection (refer to symptoms list below). The elderly and immunosuppressed may present with atypical symptoms. These patients do not have any signs of dehydration, sepsis.

- Mild pneumonia: Patient with pneumonia (Infiltrate on imaging + clinical symptoms consistent with pneumonia) and no signs of severe pneumonia (PSI > 130 or SIRS ≥ 2).

- Severe pneumonia: Fever or suspected respiratory infection, plus one of respiratory rate >30 breaths/min, severe respiratory distress, or SpO2 <90% on room air.

- Sepsis: Life-threatening organ dysfunction caused by a dysregulated host response to suspected or proven infection, with organ dysfunction. Signs of organ dysfunction including altered mental status, difficult or fast breathing, low oxygen saturation, reduced urine output, fast heart rate, weak pulse, cold extremities or low blood pressure, skin mottling, or laboratory evidence of coagulopathy, thrombocytopenia, acidosis, high lactate or hyperbilirubinemia.

- Septic shock: Persistent hypotension despite volume resuscitation, requiring vasopressors to maintain MAP ≥65 mmHg and serum lactate level >2 mmol/L.

Stratification Risk Factors by Age, Gender and Comorbidities

- Age ≥ 60 years and older
- Male gender
- Those with comorbidities (10.5% for cardiovascular disease, 7.3% for diabetes, 6.3% for chronic respiratory disease, 6% for hypertension, and 5.6% for cancer) Plus smokers.
- Immunocompromised hosts (T-cell depleted medications; Corticosteroid equivalent to prednisone >15 mg per day for more than 2 months).

Stratification by Clinical Symptoms:

- Fever
- Cough (usually dry)
- Dyspnea
- Myalgia
- Fatigue
- Anorexia
- Sore throat
- Approximately 90% of patients present with more than one symptom, and 15% of patients present with fever, cough, and dyspnea
- Patients may present with nausea or diarrhea 1 to 2 days prior to onset of fever and breathing difficulties.

Stratification based on Lab/Imaging Abnormalities[3]

- lymphocytopenia and leukopenia
- Thrombocytopenia
- Elevated CRP (>40)
- Troponin Leak
- Acute Kidney Injury (rising creatinine)
- Elevated D-Dimer
- Elevated Ferritin
- SOFA
- Infiltrate on CXR

Initial investigations:[4]

Asymptomatic patients will only need CBC, Metabolic panel, CRP, Flu/RSV PCR , Baseline CXR , baseline ECG and G6PD level.

Order the following investigations in patients with pneumonia/ICU:

Pulse oximetry	ABG (if indicated)	CBC with differential
Comprehensive metabolic panel	Coagulation screen + D-Dimer	Inflammatory markers (procalcitonin and CRP)
Serum troponin	Serum Vitamin D	LDH
CK	MRSA nasal screening[5]	Blood and Sputum Culture
Respiratory viral panel	Pneumonia PCR panel	G6PD
Ferritin	ECG (Establish QTc baseline)	CXR
Triglycerides	IL-6	

Management principles of Uncomplicated Illness + all other patients :

- Adhere to infection prevention measures.
- Vitamin C 1000 mg Tablet Daily[6].
- Zinc Acétate Lozenges one every 6-8 hours[7] .
- In patients with serum Vitamin D deficiency, supplement with Vitamin D 10,000 IU daily[8].
- If the patient meets criteria for high risk (see appendix), consult Infectious Diseases.

General Management of COVID-19 Pneumonia

- In All patients requiring assisted ventilation, placement in airborne isolation is preferred.
- All patients admitted to ICU, placement in airborne isolation is preferred.
- Airborne precaution is required for all patients undergoing aerosol generating medical procedures, including nebulizer treatment. In one study, COVID19 viral particles remained viable in aerosols for 3 hours following nebulization[9].
- If inhaler medications are needed, use MDI + spacer device.

Antimicrobial Management of COVID-19 Pneumonia

In patients < 50 yo with no comorbidities and mild pneumonia –

1. Start Ceftriaxone or Amoxicillin-Clavulanic Acid to treat secondary bacterial infection. In PCN-allergic patients, start Moxifloxacin. Treat for 5 days only.
2. If the nasal MRSA screen is positive, consider MRSA coverage for 5 days only– Preferred agent is Teicoplanin (6 mg/kg IV q12 x 3 loading dose; then 6 mg/kg IV q daily)[10].
3. Notwithstanding influenza season in Kuwait is over, if high suspicion for co-infection with influenza, consider Oseltamivir, 75 mg PO BID x 5 days (stop if Influenza A/B PCR is negative)
4. Start Hydroxychloroquine* 400 mg PO BID on day 1; then 200 mg PO BID day 2- day 5[11-14].
5. Consult ID to consider treatment with Lopinavir-ritonavir (200 Lopinavir mg/50 mg ritonavir) 2 tabs every 12 h for 6-10 days[15]. Imperative to start treatment early. In one study late initiation of Lopinavir/ritonavir did not improve mortality rate[16].
6. Obtain CRP baseline and serially follow.
7. Ask virology lab to check cycle threshold (ct) value at the initiation of treatment and at 72 hours.
8. Obtain ECG daily.
9. Review above antibiotic regimen in 72 hours

* Rationale for use: Hydroxychloroquine is an inexpensive and generally safe drug for short term use, with few drug-drug interactions. While it is unknown if it is effective to treat COVID-19, there is a favorable risk: benefit and cost ratio. Multiple trials are ongoing, and this recommendation will be updated when further data is available.
The recent French study[13] showing combination of Hydroxychloroquine and Azithromycin achieved viral suppression is methodologically weak and has not been subjected to peer review. When limiting the analysis to those with comparable baseline cycle threshold values, combination therapy with hydroxychloroquine and azithromycin led to a similar proportion of negative testing by day 6 compared to hydroxychloroquine monotherapy. Furthermore, the study does not report the clinical outcomes of these patients, and it is unknown if reductions in viral load correlate with improvements in clinical outcomes. Thus, based on this limited (only 6 patients in combination group) and weak evidence, we recommend against the routine use of azithromycin for the treatment of COVID-19 at this time.

In patients <50 yo with comorbidities OR > 50 yo regardless of associated comorbidities, and mild pneumonia:

1. If no hospitalization in the past 90 days, start Ceftriaxone or Amoxicillin-Clavulanic Acid to treat secondary bacterial infection. In PCN-allergic patients, start Moxifloxacin. Treat for 5-7 days only. If hospitalized in the past 90 days – consider piperacillin-tazobactam 4.5 mg IV q8. If PCN allergic, consider Levofloxacin 500 mg IV/PO daily. Treat for 5-7 days only.
2. If the nasal MRSA screen is positive, consider MRSA coverage for 5 days only– Preferred agent is Teicoplanin (6 mg/kg IV q12 x 3 loading dose; then 6 mg/kg IV q daily)[10].
2. Notwithstanding influenza season in Kuwait is over, if high suspicion for co-infection with influenza, consider Oseltamivir, 75 mg PO BID x 5 days (stop if Influenza A/B PCR is negative)
3. Start Hydroxychloroquine* 400 mg PO BID on day 1; then 200 mg PO BID day 2- day 5[11-14].
4. Consult ID to consider treatment with Lopinavir-ritonavir (200 Lopinavir mg/50 mg ritonavir) 2 tabs every 12 h for 6-10 days[15]. Imperative to start treatment early. In one study late initiation of Lopinavir/ritonavir did not improve mortality rate[16].
5. Obtain CRP baseline and serially follow.
6. Ask virology lab to check cycle threshold (ct) value at the initiation of treatment and at 72 hours.
7. Obtain ECG daily.
8. ***Review above antibiotic regimen in 72 hours***

In patients requiring ICU admission -

1. Start Piperacillin-Tazobactam; In PCN allergic patients – consult ID senior level physician.
2. If the nasal MRSA screen is positive, consider MRSA coverage for 5 days only– Preferred agent is Teicoplanin (6 mg/kg IV q12 x 3 loading dose; then 6 mg/kg IV q daily)[10].
3. Notwithstanding influenza season in Kuwait is over, if high suspicion for co-infection with influenza, consider Oseltamivir, 75 mg PO BID x 5 days (stop if Influenza A/B PCR is negative)
4. Start Hydroxychloroquine* 400 mg PO BID on day 1; then 200 mg PO BID day 2- day 5[11-14].
5. Consult ID to consider treatment with Lopinavir-ritonavir (200 Lopinavir mg/50 mg ritonavir) 2 tabs every 12 h for 6-10 days[15]. Imperative to start treatment early. In one study late initiation of Lopinavir/ritonavir did not improve mortality rate[16]
6. Obtain CRP baseline and serially follow.
7. Ask virology lab to check cycle threshold (ct) value at the initiation of treatment and at 72 hours.
8. Obtain ECG daily.
9. ***Review above antibiotic regimen in 72 hours.***

Treatment Considerations:

- **Plasma Therapy:**
  Convalescent patient plasma has been extracted and given to COVID-19 patients with severe disease[30]. The FDA has approved convalescent plasma therapy for COVID-19 patients under single-patient emergency Investigational New Drug Applications. This treatment should be only given after consultation with infectious disease and considered on a case by case basis.

- **Tocilizumab[20-21]:**
  IL-6 inhibitor; IV formulation. Preliminary clinical experience from China in COVID-19 reported benefit, however neutropenia can be long-lasting so risk of secondary infection is possible and unquantified. This treatment should be only given after consultation with infectious disease and considered on a case by case basis.

MONITORING for HYDROXYCHLOROQUINE (HCQ) and RENAL DOSE ADJUSTEMENT:.

- The estimated half-life is 40 days
- Monitor for hemolytic anemia with CBC every 2 days. Post-marketing studies suggest the risk of hemolysis is very low. It is reasonable to start hydroxychloroquine in most patients while awaiting G6PD testing.
- Recommend avoid taking hydroxychloroquine with antacids. Separate administration by at least 4 hours.
- Hydroxychloroquine can be crushed.
- Probably safe in pregnancy, but benefits should be balanced with possible risks (in consultation with ID).
- Most toxicities are associated with long-term use; Dizziness, headache, loss of appetite, nausea, vomiting; LFT abnormalities; Retinopathy with prolonged use (>5 years), not in the acute setting.
- CrCL <10 and hemodialysis: reduce dose to 400mg po x 1 day then 200mg po OD.
- Risk of QT prolongation: should be used with caution if other QT prolonging agent such as azithromycin or fluoroquinolones or if electrolytic imbalances (Keep potassium > 4.0 mg/dL and Magnesium > 0.82 mmol/L).
- We do not recommend co-administration with azithromycin.

- Cardiac monitoring guidance
    - Obtain baseline EKG
    - If on telemetry, check QTc and see if that corresponds to EKG QTc –If yes, use telemetry for further QTc monitoring. Otherwise Get EKG#2 and daily EKG as noted below
    - Discontinue all other QT prolonging agents
    - Do not start Hydroxychloroquine if baseline QTc > 500 msec (or QTc > 550 msec in wide QRS patients) (or) discuss with cardiology if benefit vs risk is deemed high
    - Be cautious if Baseline QTc > 470 msec (or QTc>520 msec in wide QRS patients)
- Check Telemetry QTc/ Acquire EKG#2 – preferably >2 hours after the 2nd dose of 400 mg Hydroxychloroquine
- If QTc increases by less <50 msec; and if absolute QTc < 500 msec (<550 in wide QRS) – use lower dose.
- If QTc increases by >50 msec; or if absolute QTc > 500 msec (>550 in wide QRS) - use lower dose and recheck EKG daily for 2 days.
- Any evidence of Torsades on Tele -- D/c Hydroxychloroquine regardless of QT interval.
- Note - * Wide QRS defined as QRS > 120 msec

Monitoring Parameters for Lopinavir-ritonavir (**Kaletra**):

- Alternative therapy if hydroxychloroquine is unavailable or if the patient has contraindications or adverse effects.
- Adverse events: Hepatotoxicity, pancreatitis, diabetes, QT prolongation, lipid elevations, and fat redistribution
- Major substrate and inhibitor of Cytochrome P450, and can cause severe drug-drug interactions. Thorough evaluation of a patient's medication profile and clinical pharmacy consultation should be initiated before starting therapy.
- Pregnancy: Lopinavir-ritonavir is safe to use during pregnancy.

**Not Recommended (alphabetical order): Risk/benefit ratio does not favor use**
**Angiotensin/RAS Blocking Agents (ACEi/ARBs)**[17]<br>Do not discontinue these therapies for COVID-19 disease. Multiple professional societies in cardiology and have reviewed the current data and conclude that the evidence suggesting discontinuation of ACEi/ARB therapy to decrease risk for more severe COVID-19 is not well supported at this time.<br><br>**Azithromycin**[12]<br>No activity for SARS-CoV-2. Single study of combination therapy with hydroxychloroquine does not convincingly suggest added benefit to azithromycin combination therapy, given multiple study limitations and concern for antibiotic overuse.<br><br>**Ibuprofen/NSAIDs**[18]<br>Do not discontinue these therapies for COVID-19 disease. Paracetamol is the preferred fever reducer for use in COVID-19. Although there has been theoretical concern raised for these agents worsening outcomes, no data currently exist to support this. FDA is not aware of scientific evidence connecting the use of NSAIDs, like ibuprofen, with worsening COVID-19 symptoms. The agency is investigating this issue further and will communicate publicly when more information is available.<br><br>**Corticosteroids**[19]<br>There is significant interest and controversy surrounding the role of corticosteroids for the management of severe pneumonia due to coronaviruses. The potential benefit of these agents to blunt the

inflammatory cascade seen in severe disease needs to be carefully weighed against the concerns for secondary infections, adverse events, and other complications of corticosteroid therapy. The data for corticosteroids are inconsistent, confusing, and inconclusive. While target patients where corticosteroids will improve outcomes may exist (e.g., those with cytokine-related lung injury who may develop rapidly progressive pneumonia), that population remains ill-defined. Clinicians need to carefully weigh the risks and benefits of corticosteroids on the individual patient level. This need for a risk benefit assessment in individual patients and careful consideration of dose is exemplified in the COVID-19 Diagnosis and Treatment Guide from the National Health Commission of the People's Republic of China where the authors state "***Based on respiratory distress and chest imaging***, ***may consider glucocorticoid that is equivalent to methylprednisolone 1-2 mg/kg/day for 3-5 days or less***. ***Note that large-dose glucocorticoid suppresses immune system and could delay clearance of SARS-CoV-2***." A recent consensus statement from the Chinese Thoracic Society recommends a lower dose, ≤0.5-1 mg/kg/day methylprednisolone for ≤ 7 days in select patients, after careful consideration of risks and benefits.

Discharge Criteria for Hospitalized Patients with COVID19[22]:

1. Resolution of symptoms (afebrile for more than 72 hours; pulse ox ≥94% on RA)
2. Days since testing positive being 14 days .
3. Negative real time PCR results from, at least, two consecutive sets of nasopharyngeal and throat swabs collected more than 24 hours apart (a total of 4 negative specimens). **Testing suggested to be done on Day 12 and 13 of admission to complete the 14 days of hospitalization.**
4. Improving/Regression of abnormalities on imaging.
5. Discharged patients to be home quarantine imperatively for 14 days (wear a mask at home; reduce contact with family members, particularly elderly; resides in a single room with restroom and with good ventilation) and maintain health monitoring with follow up visit in COVID19 Clinic (Jaber Hospital) in 2 weeks.
6. For more information, please follow public health guidance for home quarantine.

Appendix:

FLOW SHEET FOR STRATIFYING RISK FACTOR TO INITIATE INFECTIOUS DISEASES REFERRAL[23-28]

Clinical syndromes associated with 2019-nCoV infection:

Please check one:

- Uncomplicated illness____
- Mild pneumonia____
- Severe pneumonia____
- Sepsis. Septic shock____

Stratification Risk Factors by Age, Gender and Comorbidities

Please check all applicable:

- Age ≥ 60 years and older____
- Male gender____
- Any cardiovascular disease____
- Diabetes____
- Chronic respiratory disease____
- Hypertension____
- Smoker____
- Active malignancy____
- Immunocompromised hosts (T-cell depleted medications; Corticosteroid equivalent to prednisone >15 mg per day for more than 2 months)_____

Stratification by Clinical Symptoms

Please check all applicable:

- Fever___
- Cough (usually dry)____
- Dyspnea____
- Myalgia ___
- Rhinorrhea
- Fatigue____
- Anorexia____
- Sore throat____
- Nausea___
- Diarrhea____

Stratification based on Lab/Imaging Abnormalities

Please check all applicable:

- Lymphocytopenia ____
- Leukopenia ____
- Thrombocytopenia ____
- Elevated CRP (>40) ____
- Elevated D-Dimer ____
- Elevated Ferritin ____
- Troponin Leak____
- Acute Kidney Injury (rising creatinine) ____
- Arrhythmia ____
- Infiltrate on CXR ____

Indications for Infectious Diseases Referral:

1 **Age ≥ 70 years old**

2 **Age ≥60 plus two clinical symptoms (other than fever).**

3 **Any age plus fever on admission or at any point during hospital course**

4 **Any age plus consolidation on CXR**

5 **Any age with Lymphocytopenia ≤1.5 10^9/L**

6 **Any immunocompromised host**

High Risk Features[29]:

Epidemiological Category	Vital Signs	Labs
Age > 50	Respiratory rate > 24 breaths/min	D-dimer > 1000 ng/ml
Pre-existing pulmonary disease	Heart rate > 125 beats/min	CPK > twice upper limit of normal
Chronic Kidney disease	SpO2 <90% on ambient air	CRP > 60
Diabetes with AIc > 7.6%		LDH > 345 U/L
History of hypertension		Elevated troponin
History of Cardiovascular disease		Admission absolute lymphocyte count < 1.0
Use of biologics		Ferritin > 300 ug/L
History of transplant or other immunosuppression		
All patients with HIV (regardless of CD4 count)		

Brief Overview of Agents[29]:

Agent	Classification	Target/Mechanism	Dosing	Key Toxicities
Hydroxychloroquine (Plaquenil)	Off-label	Multiple actions: prevents binding to ACE2; prevents transport in endosome; and possibly others	400 mg PO BID x 2 doses (loading) then 200 mg BID for 5 days total.	QTc prolongation;
Lopinavir/ritonavir (LPV/r or Kaletra)	Off-label	Protease inhibitor	400/100 mg BID for up to 10 days	QTc prolongation; ALT elevation; multiple drug-drug interactions

Liverpool COVID-19 Drug Interactions: http://www.covid19-druginteractions.org/

References:

1. World Health Organization (WHO). Clinical management of severe acute respiratory infection when novel coronavirus (nCoV) infection is suspected 2020 [30 January 2020]. Available from: https://www.who.int/publications-detail/clinical-management-of-severe-acute-respiratory-infection-whennovel-coronavirus-(ncov)-infection-is-suspected
2. Lancet. 2020 Feb 15;395(10223):507-513.
3. JAMA Intern Med. Published online March 13, 2020. doi:10.1001/jamainternmed.2020.0994
4. BMJ Best Practice. COVID-19. Last updated: March 2020. https://bestpractice.bmj.com/topics/en-us/3000168, Accessed5/3/2020.
5. Clin Infect Dis. 2018 Jun18;67(1):1-7
6. Biomed Res Int. 2018;2018:1837634.
7. JRSM Open. 2017;8(5):2054270417694291
8. BMJ. 2017 Feb 15;356:i6583. doi: 10.1136/bmj.i6583.
9. N Engl J Med. March 17, 2020. DOI: 10.1056/NEJMc2004973
10. Int J Antimicrob Agents. 2020 Mar 13:105944. doi: 10.1016/j.ijantimicag.2020.105944.
11. *Clinical Infectious Diseases*, , ciaa237, https://doi.org/10.1093/cid/ciaa237
12. International Journal of Antimicrobial Agents – In Press 17 March 2020. DOI: 1016/j.ijantimicag.2020.105949
13. Int J Antimicrob Agents. 2020 Mar 4:105932. doi: 10.1016/j.ijantimicag.2020.105932.
14. https://www.fda.gov/media/136534/download
15. Expert Opin Drug Discov. 2019 Apr; 14(4):397-412
16. N Engl J Med. March 18, 2020. DOI: 10.1056/NEJMoa2001282
17. https://www.fda.gov/drugs/drug-safety-and-availability/fda-advises-patients-use-non-steroidal-anti-inflammatory-drugs-nsaids-covid-19
18. N Engl J Med. March 30, 2020. DOI: 10.1056/NEJMsr2005760
19. Open Forum Infectious Diseases, ofaa105, https://doi.org/10.1093/ofid/ofaa105 Published: 23 March 2020
20. Li Y, et al. Extraordinary GU-rich single-strand RNA identified from SARS coronavirus contributes an excessive innate immune response. Microbes Infect. 2013 Feb;15(2):88-95. https://www.ncbi.nlm.nih.gov/pubmed/23123977
21. Xiaoling X, et al. Effective treatment of Severe COVID-19 Patients with Tocilizumab. [Pre-print – not peer reviewed]. http://chinaxiv.org/abs/202003.00026.
22. https://www.ecdc.europa.eu/sites/default/files/documents/COVID-19-Discharge-criteria.pdf, accessed 21/3/2020.
23. JAMA. Published online February 24, 2020. doi:10.1001/jama.2020.2648 18.
24. China CDC Weekly. 2020, 2(8): 113-122. http://weekly.chinacdc.cn/en/article/id/e53946e2-c6c4-41e9-9a9b-fea8db1a8f51
25. ACC Clinical Bulletin Focuses on Cardiac Implications of Coronavirus (COVID-19) https://www.acc.org/latest-in-cardiology/articles/2020/02/13/12/42/acc-clinical-bulletin-focuse s-on-cardiac-implications-of-coronavirus-2019-ncov
26. Lancet Respiratory Medicine article. Published: February 24, 2020. https://www.thelancet.com/pb-assets/Lancet/pdfs/S2213260020300795.pdf
27. *JAMA.* Published online February 24, 2020. doi:10.1001/jama.2020.2648 23.
28. *Intensive Care Med* (2020). https://doi.org/10.1007/s00134-020-05967-x
29. Adopted from Massachusetts General Hospital COVID-19 Treatment Guidance (version 1.0), published 17/3/2020

30. Shen C, Wang Z, Zhao F, et al. Treatment of 5 Critically Ill Patients With COVID-19 With Convalescent Plasma. *JAMA.* Published online March 27, 2020. doi:10.1001/jama.2020.4783

Members of the COVID-19 infectious disease task force:

Dr. Wasel Aladsani, Dr. Khalid Alothman, Dr. Mohammad Alghounaim, Dr. Almunther Alhasawi, Dr. Kelly Schrapp, Dr. Osama Albaqsami, Dr. Mohammed Saraya, and Dr. Ghanem Alhojailan
