# Supplementary material for: Maternal and perinatal characteristics and outcomes of pregnancies complicated with COVID-19 in Kuwait
Source: BMC Pregnancy Childbirth. 2020 Dec 2;20:754. doi: 10.1186/s12884-020-03461-2 (PMC7709095; doi:10.1186/s12884-020-03461-2)
Supplement: Supplementary file 2 — Additional file 2: Table S1. Clinical features of pregnant patients with COVID-19 in ICU. Table S2. Clinical features of pregnant COVID-19 patients who had a miscarriage. Table S3. Clinical features of COVID-19 positive neonate. [file 12884_2020_3461_MOESM2_ESM.docx]

**Supplementary files:**

**Table S1. Clinical features of pregnant patients with COVID-19 in ICU**

|  | **Patient 1** | **Patient 2** |
| --- | --- | --- |
| Age | 33 | 39 |
| Parity | 0 | 1 |
| Comorbidities  Presenting symptoms | GDM on insulin  Fever for 2 days | Healthy  Fever and cough for 4 days |
| Gestational age at diagnosis, weeks | 38+2 | 37 |
| Mode of delivery | Emergency C/S (failure to progress) | Emergency c/s due to maternal hypoxemia |
| Time of ICU admission | 24 hours after delivery | Immediately post-delivery |
| Cause of ICU admission | Respiratory distress | Hypoxemia |
| ARDS-Berlin score | Moderate | Moderate |
| qSOFA | 2 | 3 |
| Maximum respiratory support | Intubated | Intubated |
| ECMO | 0 | 0 |
| Laboratory findings (on ICU admission)  White blood cell x 10^9^  Lymphocyte x 10^9^  Neutrophil x10^9^  Hemoglobin (g/dl)  Platelets x10^9^  C-reactive protein (mg/L)  D-Dimer (ng/mL)  Alanine transaminase (U/L)  Aspartate transaminase (U/L)  Lactate dehydrogenase (IU/L) | 4.8  0.7  3.6  123  118  200  774  14  36  552 | 18.4  1.1  16.4  115  393  19.6  906  11  31  356 |
| Duration of intubation, days | 3 | 6 |
| Duration of ICU stay, days | 5 | 12 |
| Duration of hospitalization, days | 15 | 20 |
| ARDS: Acute respiratory distress syndrome, qSOFA: quick Sequential Failure Assessment, ECMO: extracorporeal membrane oxygenation, GDM: Gestational Diabetes Mellitus  ARDS-Berlin score:Acute respiratory distress syndrome (ARDS) is classified into three stages i.e., mild, moderate, and severe, according to oxygenation severity at the onset. | | |

**Table S2. Clinical features of pregnant COVID-19 patients who had a miscarriage**

|  | **Patient 1** | **Patient 2** | **Patient 3** |
| --- | --- | --- | --- |
| Maternal age, years | 37 | 29 | 31 |
| Parity | 1 | 1 | 2 |
| Past Obstetrical history | 0 | 0 | 0 |
| Pre-existing comorbidities | 0 | 0 | 0 |
| Gestational Diabetes | 0 | 0 | 0 |
| PIH | 0 | 0 | 0 |
| Outcome | Miscarriage | Miscarriage | Miscarriage |
| Gestational age at diagnosis, weeks | 14 | 13 | 14 |
| Symptoms at diagnosis  Duration of symptoms prior to diagnosis | Sore throat  Malaise/fatigue  5 days | Runny nose  1 day | Fever  Cough  2 days |
| Total WBCx 10^9^ | 2.3 | 6.5 | 4.5 |
| Neutrophilx 10^9^ | 0.67 | 4.1 | 3.4 |
| Lymphocytesx 10^9^ | 1.5 | 1.4 | 0.8 |
| Hemoglobin (g/L) | 82 | 120 | 133 |
| Platelets x 10^9^ | 67 | 212 | 287 |
| LDH (IU/L) | Not available | 112 | 181 |
| CRP (mg/dl) | 48 | 22 | 75 |
| INR, ratio | 1.2 | 1.27 | 1.25 |
| PT, seconds | 17.8 | 17.6 | 17 |
| D-Dimer (ng/ml) | Not available | 452 | 532 |
| ALT (U/L) | 14 | 22 | 11 |
| ALP (U/L) | 68 | 39 | 39 |
| Albumin (g/L) | 39 | 39 | 35 |
| PIH: Pregnancy-induced hypertension, Total WBC: Total white blood cells, LDH: Lactate Dehydrogenase, CRP: C-Reactive Protein, INR: International Normalized Ratio, PT: Prothrombin Time, ALP: Alkaline Phosphatase | | | |

**Table S3. Clinical features of COVID-19 positive neonate**

| **Characteristics** | **Neonate 1** | **Neonate 2** |
| --- | --- | --- |
| Maternal age | 33 | 30 |
| Maternal symptoms | Fever intrapartum | Fever and sore throat |
| Interval between maternal diagnosis and delivery | 6 hours | 1 day |
| PROM | 5 days | 24 hours |
| Mode of delivery | Vaginal delivery | Vaginal delivery |
| GA at delivery | 31 | 39 |
| 1, 5 minutes Apgar score | 8/9 | 8/9 |
| Birth weight, grams | 1550 | 3215 |
| Day 5 swab | Positive | Positive |
| Day 14 swab | Negative | Positive |
| Chest x-ray | Normal | Bilateral infiltration |
| WBC, x 10^9^  Reference range 5-19 | 17.3 | 11.5 |
| Neutrophil, x10^9^  Reference range 1-5 | 5.1 | 7.9 |
| Lymphocyte  Reference range 4-11 | 10.8 | 1.4 |
| Hemoglobin (g/L)  Reference range 149-237 | 145 | 186 |
| Platelets x 10^9^  Reference range 150-400 | 417 | 278 |
| INR, ratio  Reference range 0.8-1.4 | 0.9 | 1.4 |
| PTT, seconds  Reference range 24.6-36.6 | 38 | 44 |
| Prothrombin time, seconds  Reference range 13.2-16.4 | 12 | 19.5 |
| CRP (mg/dl)  Reference range 0-8 | 0.14 | 1.1 |
| AST (U/L)  10-42 | 34 | 37 |
| ALT (U/L)  10-60 | 8 | 10 |
| ALP 48-406 (U/L) | 382 | 353 |
| LDH (IU/L) | 574 | 576 |
| Outcome | Discharged | Discharged |
| PROM: Prolonged rupture of membrane, GA at delivery: Gestational Age at delivery, WBC: White Blood Cells, PTT: Partial Thromboplastin Time | | |
